# ProCAT: a data analysis approach for protein microarrays

**DOI:** 10.1186/gb-2006-7-11-r110

**Published:** 2006-11-16

**Authors:** Xiaowei Zhu, Mark Gerstein, Michael Snyder

**Affiliations:** 1 Program in Computational Biology and Bioinformatics, Yale University, New Haven, CT 06511, USA; 2 Department of Molecular Biophysics and Biochemistry, Yale University, New Haven, CT 06511, USA; 3 Department of Computer Science, Yale University, New Haven, CT 06511, USA; 4 Department of Molecular, Cellular and Developmental Biology, Yale University, New Haven, CT 06511, USA

## Abstract

Protein microarrays provide a versatile method for the analysis of many protein biochemical activities. Existing DNA microarray analytical methods do not translate to protein microarrays due to differences between the technologies. Here we report a new approach, ProCAT, which corrects for background bias and spatial artifacts, identifies significant signals, filters nonspecific spots, and normalizes the resulting signal to protein abundance. ProCAT provides a powerful and flexible new approach for analyzing many types of protein microarrays.

## Abstract 2

ProCAT, a powerful and flexible new approach for analyzing many types of protein microarrays, is described.

## Background

DNA microarray technologies have proven to be extremely valuable for probing biological processes by measuring mRNA expression profiles. However, studies at the protein level have the potential to provide more direct information since most genes function through their protein products. Traditional investigations focus on individual proteins in a system and then combine such individual analyses to provide a more global perspective. Recently, technologies to analyze proteins in a high throughput and unbiased fashion have become feasible [1]. One particular powerful technology is protein microarrays, which contain a high density of proteins and allow a systematic probing of biochemical activities [2,3].

There are two types of protein microarrays [3]. A 'functional protein microarray' contains a set of proteins individually produced and positioned in an addressable format on a microarray surface. Functional protein microarrays are useful for identifying binding activities or targets of modification enzymes. The first version of a proteome microarray was reported in 2001 and contained 5,800 yeast proteins with amino-terminal glutathione S-transferase (GST) tags printed on the array [4]. A second version of yeast protein microarrays was generated recently and contained 5,600 proteins with carboxy-terminal 6His-HA-ZZ domain tags [5]. Proteins from both collections were overexpressed, purified and spotted onto the protein microarrays. Global proteome studies were performed on these chips to understand various biological mechanisms. For example, 87 yeast kinases were examined for their substrates using yeast protein microarrays and over 4,200 *in vitro *substrates representing 1,325 unique proteins were identified [6]. Compared with the approximately 150 known *in vivo *kinase-substrate interactions, this global study served as an important first step for dissecting yeast signaling networks. In addition to searching for kinase substrates, proteome chips can be probed with labeled proteins, DNA, lipids, antibodies and many other molecules to search for interacting proteins [4,7,8]. Large amounts of data have been generated using protein microarrays, presenting significant challenges in developing robust methods to process the raw data and building reasonable biological hypotheses from the datasets.

The second type of protein microarray, the 'analytical protein microarray' or 'antibody microarray', shares similarities with immunoassays and uses antibodies to detect specific probes. Studies have shown that these antibody arrays can recognize specific targets and generate dose-dependent signal intensities, indicating that they can be used to quantify levels of various targets in a crude mixture [9,10]. Because of the cross-reactivity of certain antibodies with a variety of proteins, only highly specific antibodies are suitable for this type of study. This remains a limiting factor in preparing antibody microarrays.

Both DNA and protein microarrays are prone to systematic errors that are usually generated from different sources, such as surface defects and spatial artifacts. Many studies have offered insight on noise subtraction in DNA microarrays [11-14], but little investigation has been done for protein microarrays. Functional protein microarrays differ in many respects from DNA microarrays. First, the goals of these two microarrays are different. DNA microarrays measure the relative DNA levels in a pool of probes, whereas functional protein arrays often aim at discovering global interactions of a single probe molecule. Second, a typical DNA microarray experiment measures signal ratios between two color channels, one for a tested mRNA sample and the other for a reference sample [15]. Signals in the second channel may serve as intrinsic controls that can help to decrease the effects of various amounts of reagent on the arrays and any local array nonuniformity. Furthermore, many current scaling methods are then based on the assumption that signal intensities should be balanced between the two color channels despite variation in slide location, intensity and other sources of systematic variation [16-18]. However, such controls are missing in one-color-channel protein microarrays. Third, several scaling approaches in DNA microarrays are based on a set of 'housekeeping' genes that give constant signal intensities at different conditions [19,20]. However, in protein microarrays, such a control group must be customized according to the type of activities that are assayed, and, therefore, a ubiquitous reference group does not exist. Fourth, unlike DNA microarrays, in which non-specific binding can often be addressed by signal comparison with mismatch probes [21], cross-reactivities of protein microarrays can not be as directly corrected for. A separate slide is, therefore, often required to be probed in parallel as a negative control in protein microarray experiments. Finally, several protein-specific artifacts serve as common noise sources in protein microarrays. In the kinase assay, for example, the signal from strongly phosphorylated spots can bleed into neighboring spots, leading to incorrect background measurement. These differences are particularly applicable to functional protein microarrays in comparison to antibody arrays, and, therefore, the normalization techniques used for DNA microarrays are usually not directly applicable to functional protein microarrays.

We have developed a new protein chip analysis tool (ProCAT) to deal with various artifacts specific to functional protein microarrays. The work started from a careful survey and characterization of all potential sources of systematic errors in protein microarrays. Specific approaches were then designed to deal with each type of noise. A correction approach is applied to reduce measurement errors in the background signals. In addition, spatial variations can be reduced efficiently through a novel two-parameter signal normalization approach and calling positive spots locally. After generating a list of positives, negative control slides are analyzed in the same approach and spots are subtracted from the list if they appear in the control slide. Slide features with poor signal qualities are also removed. Finally, signal intensities of the positives are normalized according to their protein amounts. All modules that account for the challenges in data processing specific to protein microarrays are built into ProCAT and tested.

## Results

### Overall scheme

**Figure 1:**
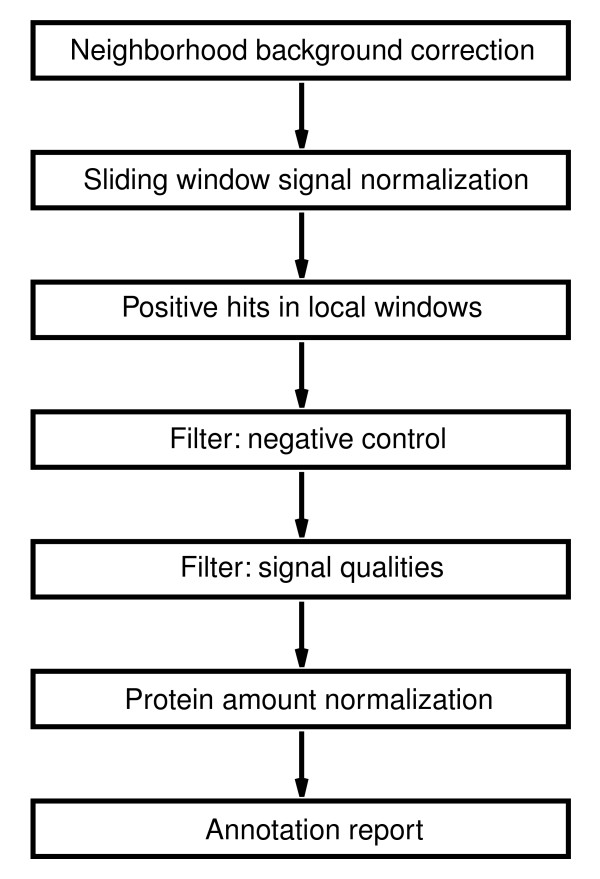
Flowchart of ProCAT. Six modules for reduction of specific array artifacts plus a report annotation module are implemented in order in the current version of ProCAT. The modular design and flexible stringencies allow the application of this approach to different functional protein microarray experiments.

ProCAT contains a flexible modular design whose individual components can be adjusted according to the experimental designs and stringency level selected by the users. Six sequential modules are currently implemented in ProCAT before a final annotation report is assembled (Figure 1). These modules carry out: background correction; signal normalization; positive spot identification; spot cross-reactivity filter; signal qualities inspection; and protein amount normalization. The performance of many of the steps was tested using several types of experiments as described below.

### Module 1: background corrections to reduce smear contaminations

**Figure 2:**
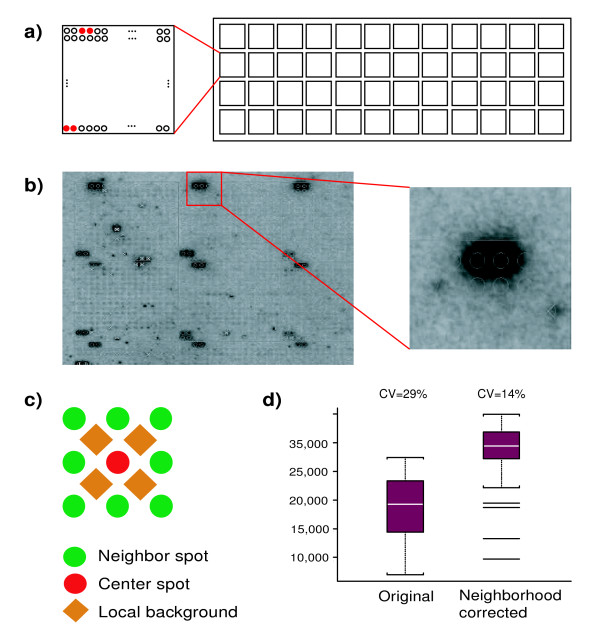
Background correction. **(a) **The test slide has an array of 4 by 12 blocks consisting of 2 pairs of positive controls (PKA) and random yeast proteins in the remaining spots in each block. **(b) **The autophosphorylation experiment showed typical bleeding problems in positive control spots. **(c) **Signal for one spot is measured as foreground minus local background intensity; therefore, artifacts in background add noise to the signal intensity. **(d) **Comparison of signal distributions of PKA spots before and after background corrections. The median of PKA signals is enhanced by 53% and the variance among the PKA spots is decreased by 41%.

A fundamental issue in all microarray experiments is background correction, which aims at reducing noise in background quantification. Signal intensities are generally quantified by subtracting the foreground intensities with the local background intensities, which are measured as the background signals immediately surrounding the spot of interest (termed here the 'adjacent background'; Figure 2b). However, in protein microarrays local background regions can be easily skewed by artifacts such as small speckles. In addition, strong positive signals from on-chip kinase assays tend to produce signal smears on both film and phosphoimagers that exceed the normal feature size (Figure 2a). In both cases, the measurement for that spot will be inaccurate. First, the background intensity will be arbitrarily high, which will diminish the real signal intensity for that spot. Second, the intensities will be affected by the alignment of the grid and extent of the smear, and, therefore, the variance of the same protein at replicate experiments will be increased.

Two methods can reduce the artifacts in local background. The user can manually adjust the grid size to fit the circles to each individual spot. However, the aligning process requires considerable time and effort. The size of the smear may even prevent refitting the grid without adversely affecting neighboring spots. Additionally, a larger spot size can diminish the signal of the spot because the signal density decreases with increasing spot size. The second method for background correction, which is applied in ProCAT, replaces the background intensity of the central spot with the background from its local neighborhood. A three by three surrounding window is assigned to each protein spot, and the median background of the nine spots will be used as the 'neighborhood background' value for the central spot (see Materials and methods for more details). No additional time is needed for further alignment, yet this method will significantly reduce artifacts that can produce erroneous measurements on spots background.

In the analysis of the phosphorylome dataset [6], we applied the neighborhood background correction and observed a high sensitivity in identifying positive targets. To further characterize the effects of neighborhood background correction, we performed a test kinase assay with 100 nM protein kinase A (PKA) spotted at 96 locations on one slide (Figure 2a). Each of the 48 blocks on the slide contains two PKA pairs with random yeast proteins spotted elsewhere (approximately 12,000 spots). After incubating the slide with ^33^P-γ-ATP, all of the PKA spots autophosphorylated and showed strong signals, and in many cases the signal went beyond the grid circle boundaries (Figure 2b). We then applied the neighborhood background correction to the PKA spots. As expected, the median for PKA signal intensities was enhanced by 53%. Furthermore, the PKA signals from different positions are more similar to each other; the variance within them is decreased by 41% (p value = 0.006; Fig 2d). Therefore, the neighborhood method for accessing background provides more robust measurements than that of the adjacent background method.

### Module 2: two-parameter signal normalization approach in sliding windows

Spatial artifacts arise from uneven signal distribution across the slide, in part due to uneven probing conditions and smear artifacts [13]. Uneven probing can occur by several means, such as uneven mixing of the probe, exposure to the probe solution, or uneven washing and drying of the slides. Two-color-channel experiments of DNA microarrays provide intrinsic controls that can be used to account for spatial artifacts. Functional protein microarrays often use only one color channel and, therefore, are especially prone to spatial artifacts. Spatial artifacts will cause inaccurate measurements of signal intensities and can hinder the identification of significant interactions. Adding more controls can help remove spatial artifacts since the signal of each spot can then be normalized according to its local controls. Due to the variable shape and size of spatial artifacts, ideally a large number of controls would be needed. However, space constraints of the protein chip and an inability to anticipate all the uses of the arrays usually prevent the necessary number of controls to fully account for spatial artifacts on the array.

**Figure 3:**
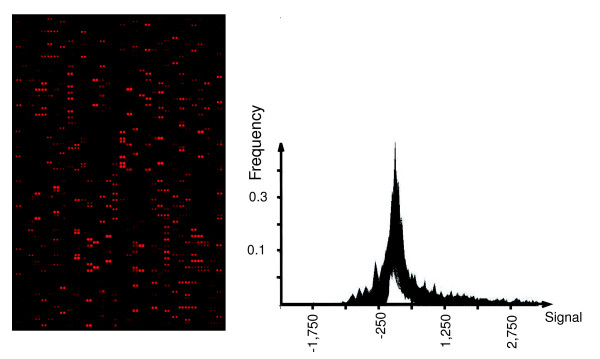
A representative protein microarray with high-quality data. The slide image was reconstructed from a protein microarray experiment with minimal noise in the data. Density plots of signals in local 37 by 37 windows (window size 9) for all spots were computationally combined, and they showed high similarities.

**Figure 4:**
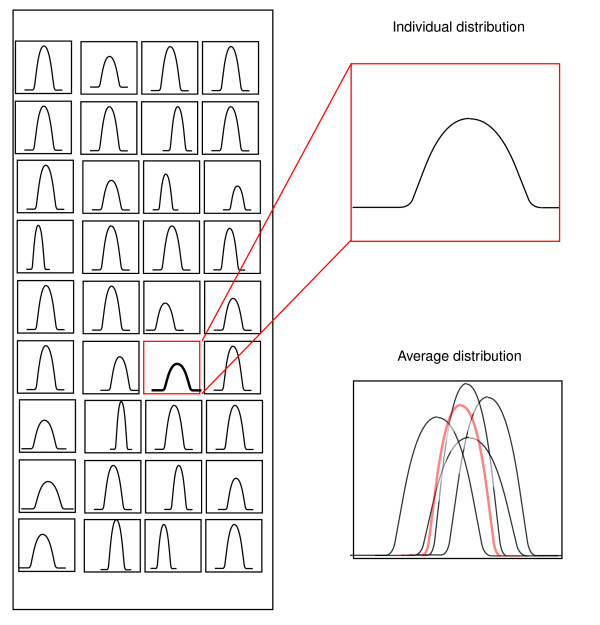
Scheme for the signal scaling method. The signal of one spot on the array is normalized according to the distribution in its local neighborhood. For each spot, a surrounding window is chosen and all spots in this window are defined as its neighborhood. The signal of a center spot will then be normalized by comparing the local median and MAD with the average values. Norm, normalized signals; Origin, original signals.

A scaling method that reduces signal variations among spots of the same proteins at different array locations decreases spatial artifacts. We developed a new normalization method to deal with the spatial artifacts specific to functional protein microarrays. By assuming that signal distribution in large windows is consistent across the slide, the foreground signal of each spot can be normalized according to signal intensities in its surrounding neighborhood. This assumption is usually valid in protein microarray experiments in which proteins are randomly printed on the array (Figure 3). Two parameters, the median and the median absolute deviation (MAD), are calculated to represent the signal distribution in the local window (Figure 4). To perform the normalization, the median and MAD of all sliding windows are averaged. The average values are then used to correct the signal of the central spot to more closely align with the global distribution of spot signals on the array (see Materials and methods for more details).

**Figure 5:**
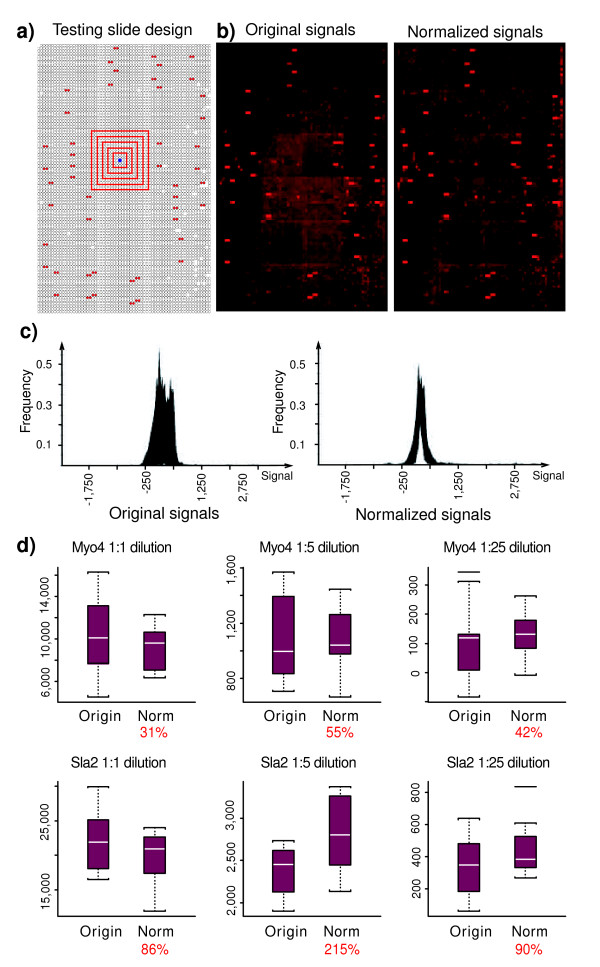
Testing experiment for the signal scaling approach. **(a) **The design of the test slide with positive spots shown as red spots and the five tested normalization window, indicated by red squares, for a given spot on the array, shown in blue. **(b) **Comparison of signal intensity before and after normalization using window size 9 on the testing experiment. The two images were computationally reconstructed from the signal files, either without or with normalization. **(c) **Density plots of signals in the local windows are shown superimposed. The distributions are more similar to each other after the signal normalization using the default window size 9. **(d) **Variation analysis for positive controls. Five out of six controls showed a decrease of variances after normalization.

To test the performance of this two-parameter scaling approach for signal normalization within one slide, we designed a test microarray containing multiple positive controls printed at different positions on the slide. The test array was organized in the same format as the commercially available protein microarrays (Invitrogen). Each protein was printed in duplicate, and the array contained 24 blocks of 16 by 16 printed proteins (Figure 5a). Two GST-fusion proteins, Sla2p and Myo4p, were purified separately and a 1:1, 1:5, and 1:25 dilution of each protein was prepared. Sla2p and Myo4p at each concentration were printed at eight random positions on the array. Other spots were occupied with bovine serum albumin (BSA) as negative controls. In order to visualize the two fusion proteins, anti-GST antibody was used to probe the slide, and one probing with typical spatial artifacts is shown in Figure 5. The artifact-containing slide showed different signal levels between the edges and the middle portion of the array. This produced blocks that had a variable signal distribution that ranged from high to low from one edge of the slide to the opposite edge; the variability occurred across blocks and simple block normalization methods adopted in DNA microarray normalization approaches [17] would not be suitable for dealing with this problem.

We applied ProCAT to normalize the slide with several different parameters (Figure 5). Five window sizes were tested, termed windows 1, 3, 5, 7, and 9. These numbers correspond to the window size as a function of the number of spots on one edge of a block. For example, a block of 20 by 20 spots analyzed using window 1 would have a window size of 0.1 that of the block edge, or in this case 2 spots above, below, and to either side of the central spot, whereas a window size of 9 would contain a 37 by 37 area roughly as large as 4 blocks. Three observations were made from the analysis of different window sizes. First, as the window size increases, the computational time used for the normalization also increases. Second, no obvious spatial artifacts were left after the normalization with any of the window sizes tested (Figure 5b). Third, a small window size diminishes any signal inequality that exists between positive signals and background noise. Indeed, a small scaling window tends to introduce extreme changes to the original signals and, therefore, increases the discrepancy between the duplicate spots of the same protein. The variance of the signals for the same protein after normalization with different window size was calculated. In five out of the six cases (three dilutions of two proteins) the scaling window 9 can successfully reduce the signal variance in a range from 31% to 90% (Figure 5c). Decrease of signal variation suggests that a large scaling window will help to reduce spatial artifacts. Although larger window sizes are possible, 9 was used as the default number for ProCAT because the analysis can be done in a reasonable time and minimal improvement has been achieved after window size 7 (Additional data file 1).

### Module 3: local window to identify positive spots

In addition to providing accurate measurements of spot intensities, ProCAT has been developed to assign thresholds for identifying positive targets in one experiment. Traditionally, a global cutoff can be calculated from all spots and applied to the whole slide. Due to variable spatial artifacts, cutoffs were assigned locally in ProCAT. For each spot on the array the signal distribution within a nine by nine window was calculated and a cutoff defined as a number of standard deviations away from the mean; the default for ProCAT is two standard deviations. This cutoff corresponds to 5% significance level if the signal distribution within this local window is normal. When many spots with strong signals are included in the window, the cutoff will be arbitrarily high and thus decrease the sensitivity of detecting positive spots by the program. To avoid this loss in sensitivity, ProCAT has a built in function to identify possible outliers, to remove those outlier spots that have extremely strong signals, and then to calculate a cutoff for identifying positive spots using the remaining spots.

**Figure 6:**
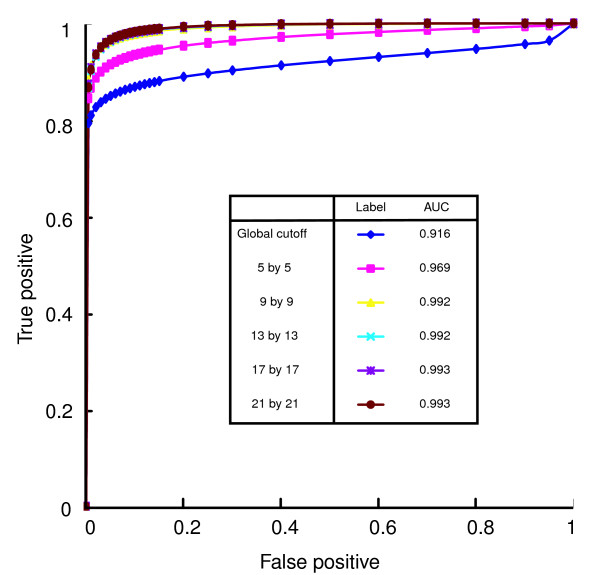
ROC curve comparing the global cutoffs and local cutoffs in calling positive spots. The test slide has six unique positive controls (Sla2p and Myo4p in three different titrations). The performance of identifying the positive controls is increased by using local cutoffs generated in relatively large surrounding windows. Five window sizes were tested and the best performance was achieved using nine by nine or larger windows.

A receiver operating characteristic (ROC) curve was used to compare the performance of local window cutoffs versus a global cutoff on the test slide [22]. Area under ROC curve (AUC) is a performance indicator that ranges from 0 to 1, with 1 for the best performing method. Using GST-Sla2p and GST-Myo4p as positive controls and BSA as negative controls, the sensitivity and specificity for both local and global cutoff methods was estimated. Five window sizes were tested and compared with the global cutoff (Figure 6). Prediction performance is increased significantly when using local windows with nine or more spots on one edge. Thus, a nine by nine window is used as the default in ProCAT since a larger window size results in increased computing time with only minimal improvement in sensitivity. The AUC value is much larger in local cutoffs (0.992) compared to global cutoffs (0.916) and the improvement is unlikely to be due to random chance (p value = 0.002) [20]. Therefore, we can conclude that the local cutoff is significantly better in identifying positive spots than a global cutoff.

### Module 4, 5: filter module; negative control and quality control as filters

Two layers of filters are implemented in ProCAT. First, all positive spots from negative control experiments are removed. For example, in on-chip kinase assays, kinase dead alleles were probed on separate arrays using the same experimental conditions as used with wild-type kinases. Spots that produce signals in the absence of active kinase were identified by ProCAT and removed from the target lists of kinase probings. When probing tagged protein to detect protein-protein interaction, testing the epitope tag in the absence of the protein of interest is also an essential control. If proper negative control experiments are available, ProCAT will analyze them in the same way as regular experiments to construct experimental positive spot lists void of proteins producing positive signals under control conditions.

The second filter checks the quality of each positive spot. All proteins are spotted in duplicates on protein microarrays, hence should have very similar signal intensities. ProCAT then uses the difference between duplicate signals as an indicator of the signal qualities. The difference between signals of two duplicate spots (*s*_1_, *s*_2_) is calculated as (*s*_1 _+ *s*_2_)/(|*s*_1_| + |*s*_2_|) and then fitted to a normal distribution. Proteins with exceptionally large differences in their duplicate spots are more likely to be biased by certain artifacts, and thus are removed from the positive list. The default threshold for the duplicate spot difference in ProCAT is set at two standard deviations away from the mean.

### Module 6: protein amount normalization

One of the goals for protein microarray experiments is to identify the affinity of a binding interaction (in a protein-protein interaction assay) or the extent of phosphorylation (in a kinase assay) so that one can compare the relative strength of the reaction for each positive protein. Ideally, the spot intensity would directly correspond to the strength of interaction. However, a number of other factors contribute to the array signal intensities, including the systematic noise from various artifacts, as was already discussed, and the amount of protein printed on the chip. Nonetheless, semi-quantitative estimates can be obtained. After background correction and signal normalization, the raw signals can be standardized by relative protein amounts before they can be used to estimate the interaction strength.

Although proteins on the microarray can have very different amounts, they do share the same epitopes for the purpose of large-scale protein purification [4,5]. Therefore, probing with anti-epitope antibodies will provide an estimate of the relative protein amounts in each spot on the array. After the protein amount is determined for one spot at row *i *and column *j*, ProCAT divides the raw signal intensities *S*_*i*,*j *_by the protein amount signals *A*_*i*,*j *_and uses the quotient as an approximation of the strengths of interactions:

*I*_*i*,*j *_= *S*_*i*,*j*_/*A*_*i*,*j*_

This approximation generally works well across the slide except for the following two situations. Less abundant proteins will be biased because the *A*_*i*,*j *_values estimated in anti-epitope probings are more susceptible to background noise and slide artifacts. On the other hand, overpowering spots can also be biased if they have saturated signal intensities. A saturated *S*_*i*,*j *_value is an underestimate to the real signal. For these two reasons, only proteins with amounts more than a minimal cutoff and signal intensities lower than a saturation threshold will be normalized with protein amounts. Proteins that do not conform to these two requirements will be recorded with unnormalized signals and flagged for further inspection. An additional caveat is that the relative protein amount assessed using antibodies includes both native and denatured protein at a given spot. Therefore, the estimation of interaction strength will be an underestimate since the amount of functional protein may be an overestimate.

### ProCAT as a modular web tool

ProCAT was designed as a flexible tool to analyze functional protein microarray data. The program was scripted in Perl (version 5.6.1) on top of a Tomcat (version 5.0.30) web server [23]. Each module discussed above was implemented independently and can be included or excluded depending on various experimental designs. To input a dataset, the user has to characterize the data in three aspects: experimental designs, data file formats and normalization parameters. Experimental design contains parameters such as the number of test arrays and negative control arrays for one particular assay. Data file format describes the layout in the uploaded dataset so that ProCAT can recognize and extract the useful information from it. Normalization parameters allow users to try different stringency levels. These three levels supply sufficient information to uniquely characterize an experiment while still allowing ample flexibility for the individual user to customize parameters to suit many different types of experimental designs.

After inputting all three descriptions and uploading the dataset, ProCAT takes five minutes on average to complete all analysis modules for each array. The time may vary depending on the selected analysis modules and the size of the protein microarrays. Each task is assigned a unique ID and results are organized into a database for future queries. Processed data including analysis parameters, a list of positive spots with protein annotations, and normalized signal intensities will be available for the users to download from the server.

## Discussion

Functional protein microarrays serve as an efficient platform for screening protein biochemical functions. Here we present ProCAT as a systematic approach to process and analyze data specific to functional protein microarrays. Calibrated by explicit test experiments, ProCAT has proven to be able to handle many types of functional protein microarray studies with three unique features. ProCAT includes novel scaling methods that provide robust and reproducible measurement for quantitative signals. This is crucial for protein microarrays as chip signal intensities often indicate strength of interactions. In addition, by calling positive candidates locally, ProCAT demonstrated excellent performance in identifying positives in comparison to global thresholds. Finally, each step has been integrated into a modular design to fit various experimental designs and stringency requirements.

A major challenge in designing any automated data processing method is thinking of and anticipating all possible situations that may arise. ProCAT uses a local three by three window to correct background containing signal smears or dust speckles. This method assumes the artifacts are sparse enough so that the majority of the nine spots in the local window still provide correct measurements of the background signals. Since the median value of nine spots is used to correct the background, a few biased spots within the window will not severely affect the corrected background value. This assumption is usually valid since the percentage of spots that are either positive or whose signal is contaminated by artifacts in protein microarray experiments is generally quite low. In extreme cases where such spots are likely to be very close to each other, a larger window (five by five for example) can be used. Large artifacts such as bright speckles and incubation bubbles may affect many spots in a particular region. Since the shapes of these artifacts are variable, it is necessary to manually flag these spots initially and then remove them from future analysis. Many commercially available software packages for microarray experiments have a built in flagging function, and ProCAT will automatically discard flagged spots.

A key aspect of ProCAT is the two-parameter approach for reducing spatial nonuniformity. Several factors can affect the performance of ProCAT's normalization. First, ProCAT normalizes the signal of a spot according to the signal distribution in its local neighborhood. It diminishes the signal intensity if the spot is located in a high signal neighborhood, while compensating the intensity if it is in a low signal neighborhood. This approach is based on the assumption that signal intensities across the slide share the same distribution, and it holds true if and only if the regional variations observed on the slide are due to technical artifacts and not from real biological differences. Since proteins are printed in a random order on most of the current protein microarrays, it is unlikely a particular region of the slide will gain high intensities as a result of biologically relevant reasons. Second, the size of the neighborhood window can also largely affect the performance of the normalization. Small window sizes tend to add biases to signals and diminish all local variations, whereas large window sizes increase the computational burden and tend to preserve local variations. We found that the optimal window size of ProCAT is 9 for protein-protein interactions; this figure corresponds to approximately four blocks on the chip and is used as the default. Other window sizes can also be chosen to fit various shapes of spatial artifacts.

ProCAT can be applied to many experiments using protein microarrays, such as kinase assays, protein-protein interactions and protein-DNA interactions. Thus far, the two-parameter scaling approach has only been used in single chip normalization; however, a similar strategy can be extended to rescale multiple slides by assuming signals in neighborhood windows on different slides are similarly distributed. Overall, ProCAT provides a powerful and flexible new approach for optimal processing and analysis of functional protein microarrays.

## Materials and methods

### Preparation of the testing slide

For the slide used for testing background correction, 100 nM PKA (Sigma, St. Louis, MO, USA) was spotted at 96 different places as positive control. The slide was incubated with 200 μl of kinase buffer (100 mM Tris pH 8.0, 100 mM NaCl, 10 mM MgCl_2_, 20 mM glutathione, 20% glycerol) plus 0.5 mg/ml BSA, 0.1% Triton X-100, and 2 μl ^33^P-γ-ATP in a humidified chamber at 30°C for 1 hour. The slide was then washed twice with 10 mM Tris pH 7.4, 0.5% SDS and once with double distilled H_2_O before being spun dry and exposed to X-ray film (Kodak, Rochester, NY, USA).

For the anti-GST probing, slides were printed with Sla2p and Myo4p as positive controls and 150 nM BSA as a negative control. The array surface was blocked using SuperBlock (Pierce, Rockford, IL, USA) at 4°C for 1 hour. Rabbit polyclonal IgG (Santa Cruz Biotechnology, Santa Cruz, CA, USA) was incubated with the slides at 1,000-fold dilution. The array was then washed with PBST (Sigma) and incubated with a 1:1,000 dilution of Cy5-conjugated anti-rabbit IgG antibody (Jackson Laboratories, Bar Harbor, ME, USA). Slides were then washed with PBST five times and scanned in an Axon GenePix scanner (Molecular Devices, Sunnyvale, CA, USA). Raw signals were extracted with GenePix Pro 6.0 software (Molecular Devices).

### Signal quantification and background correction

For one spot, let *i *be the row and *j *the column on a protein microarray. Thus, *B*_*i*,*j *_represents the adjacent background intensity and *F*_*i*,*j *_denotes the foreground intensity. The raw signal intensity *S*_*i*,*j *_is calculated as:

*S*_*i*,*j *_= *F*_*i*,*j *_- *B*_*i*,*j*_

In neighborhood background correction, we use neighborhood background to replace the adjacent background. A local three by three window around *B*_*i*,*j *_is chosen and the neighborhood background B^i,j
 MathType@MTEF@5@5@+=feaafiart1ev1aaatCvAUfeBSjuyZL2yd9gzLbvyNv2Caerbhv2BYDwAHbqedmvETj2BSbqee0evGueE0jxyaibaiKI8=vI8tuQ8FMI8Gi=hEeeu0xXdbba9frFj0=OqFfea0dXdd9vqai=hGuQ8kuc9pgc9s8qqaq=dirpe0xb9q8qiLsFr0=vr0=vr0dc8meaabaqaciGacaGaaeqabaqadeqadaaakeaaceWGcbGbaKaadaWgaaWcbaGaamyAaiaacYcacaWGQbaabeaaaaa@36BD@ is defined as:

B^i,j=mediani−1≤i′≤i+1j−1≤j′≤j+1(Bi′,j′)
 MathType@MTEF@5@5@+=feaafiart1ev1aaatCvAUfeBSjuyZL2yd9gzLbvyNv2Caerbhv2BYDwAHbqedmvETj2BSbqee0evGueE0jxyaibaiKI8=vI8tuQ8FMI8Gi=hEeeu0xXdbba9frFj0=OqFfea0dXdd9vqai=hGuQ8kuc9pgc9s8qqaq=dirpe0xb9q8qiLsFr0=vr0=vr0dc8meaabaqaciGacaGaaeqabaqadeqadaaakeaaceWGcbGbaKaadaWgaaWcbaGaamyAaiaacYcacaWGQbaabeaakiabg2da9maaxababaGaamyBaiaadwgacaWGKbGaamyAaiaadggacaWGUbaalqaabeqaaiaadMgacqGHsislcaaIXaGaeyizImQabmyAayaafaGaeyizImQaamyAaiabgUcaRiaaigdaaeaacaWGQbGaeyOeI0IaaGymaiabgsMiJkqadQgagaqbaiabgsMiJkaadQgacqGHRaWkcaaIXaaaaeqaaOWaaeWaaeaacaWGcbWaaSbaaSqaaiqadMgagaqbaiaacYcaceWGQbGbauaaaeqaaaGccaGLOaGaayzkaaaaaa@55DB@

### Two-parameter signal normalization approach in sliding windows

In a protein slide with *N *rows and *M *columns, a local window *W*_*i*,*j *_around one spot (*i*, *j*) is defined as signals of a set of spots *S*_*i*,*j *_that satisfy:

*W*_*i*,*j*_(*k*) = {*S*_*i'*,*j' *_| max(1, *i *- *k*) ≤ *i' *≤ min(*N*, *i *+ *k*), max(1, *j *- *k*) ≤ *j' *≤ min(*M*, *j *+ *k*)}

The size parameter *k *is dependent on window size factor *f*_*win *_and the block size *f*_*block*_:

k=fblockfwin10
 MathType@MTEF@5@5@+=feaafiart1ev1aaatCvAUfeBSjuyZL2yd9gzLbvyNv2Caerbhv2BYDwAHbqedmvETj2BSbqee0evGueE0jxyaibaiKI8=vI8tuQ8FMI8Gi=hEeeu0xXdbba9frFj0=OqFfea0dXdd9vqai=hGuQ8kuc9pgc9s8qqaq=dirpe0xb9q8qiLsFr0=vr0=vr0dc8meaabaqaciGacaGaaeqabaqadeqadaaakeaacaWGRbGaeyypa0ZaaSaaaeaacaWGMbWaaSbaaSqaaiaadkgacaWGSbGaam4BaiaadogacaWGRbaabeaakiaadAgadaWgaaWcbaGaam4DaiaadMgacaWGUbaabeaaaOqaaiaaigdacaaIWaaaaaaa@406B@

in which *f*_*block *_represents the number of spots on one edge of the block, and *f*_*win *_is chosen by users from five options: 1, 3, 5, 7 and 9. Different windows can overlap with each other and go beyond the block edges. Let *s *denote signal intensities of spots within the local window; ProCAT uses two parameters to characterize the signal distribution of *s*: median (*MED*) and median absolute deviation (*MAD*):

MEDi,j=medians∈Wi,j(k)(s)
 MathType@MTEF@5@5@+=feaafiart1ev1aaatCvAUfeBSjuyZL2yd9gzLbvyNv2Caerbhv2BYDwAHbqedmvETj2BSbqee0evGueE0jxyaibaiKI8=vI8tuQ8FMI8Gi=hEeeu0xXdbba9frFj0=OqFfea0dXdd9vqai=hGuQ8kuc9pgc9s8qqaq=dirpe0xb9q8qiLsFr0=vr0=vr0dc8meaabaqaciGacaGaaeqabaqadeqadaaakeaacaWGnbGaamyraiaadseadaWgaaWcbaGaamyAaiaacYcacaWGQbaabeaakiabg2da9maaxababaGaamyBaiaadwgacaWGKbGaamyAaiaadggacaWGUbaaleaacaWGZbGaeyicI4Saam4vamaaBaaameaacaWGPbGaaiilaiaadQgaaeqaaSGaaiikaiaadUgacaGGPaaabeaakiaacIcacaWGZbGaaiykaaaa@49E1@

MADi,j=medians∈Wi,j(k)(|s−MEDi,j|)
 MathType@MTEF@5@5@+=feaafiart1ev1aaatCvAUfeBSjuyZL2yd9gzLbvyNv2Caerbhv2BYDwAHbqedmvETj2BSbqee0evGueE0jxyaibaiKI8=vI8tuQ8FMI8Gi=hEeeu0xXdbba9frFj0=OqFfea0dXdd9vqai=hGuQ8kuc9pgc9s8qqaq=dirpe0xb9q8qiLsFr0=vr0=vr0dc8meaabaqaciGacaGaaeqabaqadeqadaaakeaacaWGnbGaamyqaiaadseadaWgaaWcbaGaamyAaiaacYcacaWGQbaabeaakiabg2da9maaxababaGaamyBaiaadwgacaWGKbGaamyAaiaadggacaWGUbaaleaacaWGZbGaeyicI4Saam4vamaaBaaameaacaWGPbGaaiilaiaadQgaaeqaaSGaaiikaiaadUgacaGGPaaabeaakmaabmaabaWaaqWaaeaacaWGZbGaeyOeI0IaamytaiaadweacaWGebWaaSbaaSqaaiaadMgacaGGSaGaamOAaaqabaaakiaawEa7caGLiWoaaiaawIcacaGLPaaaaaa@5344@

After calculating *MED*_*i*,*j *_and *MAD*_*i*,*j *_for all the spots on the array, they are averaged to obtain the two parameters MED¯
 MathType@MTEF@5@5@+=feaafiart1ev1aaatCvAUfeBSjuyZL2yd9gzLbvyNv2Caerbhv2BYDwAHbqedmvETj2BSbqee0evGueE0jxyaibaiKI8=vI8tuQ8FMI8Gi=hEeeu0xXdbba9frFj0=OqFfea0dXdd9vqai=hGuQ8kuc9pgc9s8qqaq=dirpe0xb9q8qiLsFr0=vr0=vr0dc8meaabaqaciGacaGaaeqabaqadeqadaaakeaadaqdaaqaaiaad2eacaWGfbGaamiraaaaaaa@35A3@ and MAD¯
 MathType@MTEF@5@5@+=feaafiart1ev1aaatCvAUfeBSjuyZL2yd9gzLbvyNv2Caerbhv2BYDwAHbqedmvETj2BSbqee0evGueE0jxyaibaiKI8=vI8tuQ8FMI8Gi=hEeeu0xXdbba9frFj0=OqFfea0dXdd9vqai=hGuQ8kuc9pgc9s8qqaq=dirpe0xb9q8qiLsFr0=vr0=vr0dc8meaabaqaciGacaGaaeqabaqadeqadaaakeaadaqdaaqaaiaad2eacaWGbbGaamiraaaaaaa@359F@ for the reference distribution.

For one spot (*i*, *j*), ProCAT normalizes its raw signal *S*_*i*,*j *_by comparing *MED*_*i*,*j *_and *MAD*_*i*,*j *_with the average values:

S^i,j=MED¯+(Si,j−MEDi,j)MAD¯MADi,j
 MathType@MTEF@5@5@+=feaafiart1ev1aaatCvAUfeBSjuyZL2yd9gzLbvyNv2Caerbhv2BYDwAHbqedmvETj2BSbqee0evGueE0jxyaibaiKI8=vI8tuQ8FMI8Gi=hEeeu0xXdbba9frFj0=OqFfea0dXdd9vqai=hGuQ8kuc9pgc9s8qqaq=dirpe0xb9q8qiLsFr0=vr0=vr0dc8meaabaqaciGacaGaaeqabaqadeqadaaakeaaceWGtbGbaKaadaWgaaWcbaGaamyAaiaacYcacaWGQbaabeaakiabg2da9maanaaabaGaamytaiaadweacaWGebaaaiabgUcaRmaabmaabaGaam4uamaaBaaaleaacaWGPbGaaiilaiaadQgaaeqaaOGaeyOeI0IaamytaiaadweacaWGebWaaSbaaSqaaiaadMgacaGGSaGaamOAaaqabaaakiaawIcacaGLPaaadaWcaaqaamaanaaabaGaamytaiaadgeacaWGebaaaaqaamaanaaabaGaamytaiaadgeacaWGebWaaSbaaSqaaiaadMgacaGGSaGaamOAaaqabaaaaaaaaaa@4E1C@

### Identifying positive spots in local windows

For a given spot at row *i *and column *j*, its normalized signal S^i,j
 MathType@MTEF@5@5@+=feaafiart1ev1aaatCvAUfeBSjuyZL2yd9gzLbvyNv2Caerbhv2BYDwAHbqedmvETj2BSbqee0evGueE0jxyaibaiKI8=vI8tuQ8FMI8Gi=hEeeu0xXdbba9frFj0=OqFfea0dXdd9vqai=hGuQ8kuc9pgc9s8qqaq=dirpe0xb9q8qiLsFr0=vr0=vr0dc8meaabaqaciGacaGaaeqabaqadeqadaaakeaaceWGtbGbaKaadaWgaaWcbaGaamyAaiaacYcacaWGQbaabeaaaaa@36CE@ is compared to surrounding spots in a nine by nine window *W*_*ij*_(4). Signals within this window are fit to a normal distribution. The mean μ_*i*,*j *_and standard deviation σ_*i*,*j *_will be calculated and the default threshold is set at two standard deviations above the signal mean. A spot (*i*, *j*) will be called positive only if its signal is above the threshold:

S^i,j
 MathType@MTEF@5@5@+=feaafiart1ev1aaatCvAUfeBSjuyZL2yd9gzLbvyNv2Caerbhv2BYDwAHbqedmvETj2BSbqee0evGueE0jxyaibaiKI8=vI8tuQ8FMI8Gi=hEeeu0xXdbba9frFj0=OqFfea0dXdd9vqai=hGuQ8kuc9pgc9s8qqaq=dirpe0xb9q8qiLsFr0=vr0=vr0dc8meaabaqaciGacaGaaeqabaqadeqadaaakeaaceWGtbGbaKaadaWgaaWcbaGaamyAaiaacYcacaWGQbaabeaaaaa@36CE@ > μ_*i*,*j *_+ 2σ_*i*,*j*_

When positive spots are likely to be close to each other, ProCAT uses box plots to examine and remove possible outliers from the surrounding window [24]. Let *Q*_1 _be the lower quartile (25th percentile) and *Q*_2 _be the upper quartile (75th percentile); the difference between *Q*_1 _and *Q*_2 _is termed interquartile range Δ*Q*. A spot (*i*', *j*') is then defined as an outlier if its signal:

S^i′,j′
 MathType@MTEF@5@5@+=feaafiart1ev1aaatCvAUfeBSjuyZL2yd9gzLbvyNv2Caerbhv2BYDwAHbqedmvETj2BSbqee0evGueE0jxyaibaiKI8=vI8tuQ8FMI8Gi=hEeeu0xXdbba9frFj0=OqFfea0dXdd9vqai=hGuQ8kuc9pgc9s8qqaq=dirpe0xb9q8qiLsFr0=vr0=vr0dc8meaabaqaciGacaGaaeqabaqadeqadaaakeaaceWGtbGbaKaadaWgaaWcbaGabmyAayaafaGaaiilaiqadQgagaqbaaqabaaaaa@36E6@ > *Q*_2 _+ 1.5 Δ*Q*

or

S^i′,j′
 MathType@MTEF@5@5@+=feaafiart1ev1aaatCvAUfeBSjuyZL2yd9gzLbvyNv2Caerbhv2BYDwAHbqedmvETj2BSbqee0evGueE0jxyaibaiKI8=vI8tuQ8FMI8Gi=hEeeu0xXdbba9frFj0=OqFfea0dXdd9vqai=hGuQ8kuc9pgc9s8qqaq=dirpe0xb9q8qiLsFr0=vr0=vr0dc8meaabaqaciGacaGaaeqabaqadeqadaaakeaaceWGtbGbaKaadaWgaaWcbaGabmyAayaafaGaaiilaiqadQgagaqbaaqabaaaaa@36E6@ <*Q*_1 _– 1.5 Δ*Q*

To obtain a robust threshold, the corrected mean μ^i,j
 MathType@MTEF@5@5@+=feaafiart1ev1aaatCvAUfeBSjuyZL2yd9gzLbvyNv2Caerbhv2BYDwAHbqedmvETj2BSbqee0evGueE0jxyaibaiKI8=vI8tuQ8FMI8Gi=hEeeu0xXdbba9frFj0=OqFfea0dXdd9vqai=hGuQ8kuc9pgc9s8qqaq=dirpe0xb9q8qiLsFr0=vr0=vr0dc8meaabaqaciGacaGaaeqabaqadeqadaaakeaacuaH8oqBgaqcamaaBaaaleaacaWGPbGaaiilaiaadQgaaeqaaaaa@37AC@ and standard deviation σ^i,j
 MathType@MTEF@5@5@+=feaafiart1ev1aaatCvAUfeBSjuyZL2yd9gzLbvyNv2Caerbhv2BYDwAHbqedmvETj2BSbqee0evGueE0jxyaibaiKI8=vI8tuQ8FMI8Gi=hEeeu0xXdbba9frFj0=OqFfea0dXdd9vqai=hGuQ8kuc9pgc9s8qqaq=dirpe0xb9q8qiLsFr0=vr0=vr0dc8meaabaqaciGacaGaaeqabaqadeqadaaakeaacuaHdpWCgaqcamaaBaaaleaacaWGPbGaaiilaiaadQgaaeqaaaaa@37B9@ are generated using the non-outlier spots.

## Additional data files

The following additional data files are available with the online version of this paper. Additional data file 1 is a figure illustrating the variance reduction in positive controls using different normalization window sizes. Additional data file 2 is a table listing the raw signals generated in the autophosphorylation experiment for testing the background correction method. Additional data file 3 is a table listing the raw signals generated in the anti-GST probing experiment calibrating the signal scaling approach.

## Supplementary Material

Additional File 1 Variance reduction in positive controls using different normalization window sizes. Click here for file

Additional File 2 Raw signals generated in the autophosphorylation experiment for testing the background correction method. Click here for file

Additional File 3 Raw signals generated in the anti-GST probing experiment calibrating the signal scaling approach. Click here for file
